# Consumer Financial Protection Versus Catastrophic Healthcare Expenditure in Zambia

**DOI:** 10.1002/puh2.207

**Published:** 2024-07-02

**Authors:** MccPowell Fombang, Richard Wamalwa Wanzala

**Affiliations:** ^1^ Department of Accountancy Vaal University of Technology Johannesburg South Africa; ^2^ Johannesburg Business School University of Johannesburg Johannesburg South Africa

**Keywords:** CHE, health systems, liquidity protection, OOP health expenses, universal healthcare, Zambia

## Abstract

**Background:**

Out‐of‐pocket (OOP) expenses for healthcare are regarded as catastrophic, especially if they account for a substantial amount of a poor household's effective income. This study looks at the frequency and severity of catastrophic healthcare expenditures (CHE) to evaluate the level of monetary safeguarding provided by the present healthcare system in Zambia.

**Methods:**

The study relied on the 2014 Zambia Household Health Expenditure and Utilization Survey, which was carried out in 10 different provinces. The investigated population is divided into quintiles, which divide family units into five groups, each of which represents 20% of the population. The data were analyzed using descriptive statistics, analysis of variance, and pairwise comparisons among the quintiles.

**Results:**

At 5% level of significance, pairwise analyses of the average of OOP healthcare expenditures as a proportion of non‐food spending reveal that the quintiles have statistically different means. If 10% limit is employed, the concentration index $C_{S}$ was −0.41, whereas $C_{O}$ was −0.67. At a 10% limit, the adjusted headcount ($H_{A}$) is 0.37, and the adjusted overshoot ($O_{A}$) is 0.15%.

**Conclusion:**

The frequency and severity of CHE were negligible during the study period. However, the less fortunate are more susceptible compared to the wealthy to be subjected to the occurrence and severity of CHE. Therefore, policy changes ought to emphasize the protection of the poor and vulnerable to accomplish the goal of universal healthcare (UHC). Finally, research is suggested to include equity and quality in the use of healthcare services.

## 1 Introduction

The United Nations Sustainable Development Goals have advocated for universal health coverage (UHC), which includes having access to quality essential medical care, risk‐free financial security, and secure, high‐quality, efficient, and cost‐effective key vaccines and medicines for all [1, 2]. The World Health Organization has also advocated for UHC by encouraging the elimination of financing obstacles to medical care using advance payment and the amalgamation of health‐related funds [3]. As a result, safeguarding against the adverse effects of having to make payments for healthcare on families’ wellness has evolved into an international issue. Healthcare is mainly financed using out‐of‐pocket (OOP) spending by families in many developing countries, including sub‐Saharan Africa and Zambia in particular [4]. As a result, the question arises as to how countries like Zambia can provide universal healthcare while also protecting their patients from costly OOP expenses and cushioning them from economic ruin and poverty [5]. Consequently, this study looks at the frequency and severity of catastrophic healthcare expenditures (CHEs) to evaluate the level of monetary safeguarding provided by the present healthcare system in Zambia.

Globally, there has been a push since 1978 (e.g., the International Conference on Primary Health Care) for developing economies like Zambia, to implement free primary healthcare [6]. Due to the standard of medical services deteriorating to abysmal levels and concerns about sustainability, sub‐Saharan African countries were inspired to join the Bamako Initiative in 1987, which suggested sustainable structures for healthcare facilities and included patients’ charges levied at the point of service at a particular health facility. As a result, to raise extra income for the country's healthcare system, the Government of the Republic of Zambia (GRZ) established fees for users in Zambia in 1993 in the form of a cost‐sharing measure [7]. In a scenario where GRZ financing for welfare services was inadequate and irregular, imposing fees at medical centers produced income that facilitated service consistency and standard enhancement [7]. User fees are still waived for children under the age of 5, pregnant women, and people over 65 years of age. Vulnerable people were also barred from settling patient charges, but the criteria for determining who was vulnerable were ambiguous, and the user‐fee exclusion policies were inconsistently enforced.

Zambia experienced a one‐third drop in nationwide patients seeking treatment services at hospitals and primary healthcare centers in the initial 2 years resulting from the introduction of user charges, which was followed by slower decreases in subsequent years. According to Ref. [8], the value of income collected through user fees was also inadequate, accounting for less than 10% of the overall health spending on average. A major impediment to healthcare accessibility for those who are impoverished was identified as a serious issue regarding the enactment of patients’ charges at the main medical amenities in Zambia. Concerns about the equitable impact of charging users gave skepticism on the suitability of a user‐fee policy, leading to civil and public society pressure on Zambia to reassess it. In addition, Zambia's position as a recipient of the Highly Indebted Poor Country Initiative, which decreased the financial strain that impoverished nations like Zambia encountered in loan repayment, freed up financial resources for increased spending on public space. However, the GRZ has made public its intention to achieve UHC. The Ministry of Health is now working on a health financing plan to lead the country on an equitable path to UHC. The preparation of a health financing plan necessitates the collection of credible evidence to evaluate the up‐to‐date healthcare system and its ambitious goals. Consequently, the present research looks into the frequency and severity of CHE to assess the degree of liquidity safeguard offered by the current medical system in Zambia. The investigation also looks into whether the frequency and severity of disastrous settlements are distributed equitably between those who are wealthy and those who are not.

## 2 Empirical Review

Because of Zambia's national abolition of fees for users in 2012, no comprehensive studies addressing financial security and, in particular, the impact of CHE have yet been published. Some investigations kick off to investigate concerns regarding equity and quality in healthcare delivery, but they fail to entirely tackle these obstacles [8, 9, 10]. In Zambia, Kaonga et al. [8] used an ordered logistic regression approach to investigate the factors that influence the risk of hardship financing contingent on reporting an illness and OOP expenditure. According to their findings, up to 11% of households reporting a medical condition borrowed money, sold goods, requested a friend for help, or shifted other household consumption to cover costs for healthcare. The probability of hardship financing was greater in poorer households, female‐headed households, and households living further away from healthcare facilities. As a consequence, Kaonga et al. [8] recommended that physical access and the quality of healthcare provided by the government be improved to reduce the prevalence of hardship financing, particularly among the poorest.

Song et al. [11] used regression analysis to analyze long‐term patterns of the prevalence and extent of CHE and healthcare poverty from 1986 to 2009 and presented four major findings. Initially, between 1986 and 2002, the frequency and severity of CHE among urban Chinese households grew rapidly but then stabilized. Second, the prevalence of medical deprivation and the extent of it in the poverty gap were steady before 2002 and swiftly dropped after 2002. Third, between 1986 and 2002, revenue and geographical disparities in estimates of CHE widened. Fourth, income and regional disparities in medical poverty stayed unaltered between 1986 and 2002 but narrowed significantly after 2002. From 2013 to 2018, Chen et al. [2] investigated the magnitude and distribution of CHE. They used a multistage stratified random sampling procedure with a threshold of 25% of non‐food family spending. However, to examine the frequency and severity of CHE, the concentration index was used to assess the magnitude of disparities in CHE, and a logistical model was used to assess the sociodemographic and economic variables that influence CHE. Their study found that the frequency and severity of CHE increased between 2013 and 2018. Furthermore, households with low incomes had a higher concentration of CHE.

Sharma et al. [12] used three rounds of cross‐sectional Bhutan Living Standard Surveys conducted in 2007, 2012, and 2017 to examine the CHE and poverty. They analyzed the data using the combined measurement of financial hardship and logistic regression. Their findings show that financial hardship disproportionately affects rural residents and lower income households. According to Sataru et al. [13], distance to health facilities is a barrier to access on demand, whereas the accessibility of medicines and adequate healthcare workers are supply obstacles to access. Nevertheless, because it is an investigation that uses qualitative data, it does not measure the level of how these obstacles influence utilization or enforce additional costs for obtaining products and services.

The research relied on reviews by experts and unpublished writing and on secondary and primary data coming from each of the six countries studied: Ghana, Burkina Faso, Liberia, Burundi, Senegal, and Uganda. The review indicates that, aside from fee reductions, the countries under examination have made minimal attempts to deal with both supply‐ and demand‐side obstacles to access. Another significant result concerns purchasing arrangements with providers. The abolition of user fees necessitates making plans to compensate establishments for their revenue loss. Where establishments originally retained income and employed it to enhance the delivery of services, the requirement to compensate establishments becomes even more pressing.

## 3 Methods

### 3.1 Data Sources

The Zambia Household Health Expenditure and Utilization Survey (ZHHEUS) data were used for this study (data are available at: https://www.dhsprogram.com/pubs/pdf/fr304/fr304.pdf). The ZHHEUS was a nationwide household survey conducted in 2014 by the University of Zambia, the Zambian Ministry of Health, and the Central Statistics Office [14]. This study used a comprehensive guide formulated by Wagstaff et al. [15] to analyze ZHHEUS. In the guide, the proportion of OOP in the overall spending establishes whether or not CHE occurred. The more often CHE occurs above the predetermined limit, the higher the severity of CHE. The approach outlined below is meant for estimating the country's frequency and severity of CHE. The research investigation begins by estimating the frequency and severity of CHE, assuming that CHE has been equally adjusted irrespective of who suffers from it. The investigation subsequently estimates a distribution‐sensitive frequency and severity of CHE by giving greater significance to CHE spent with those in poverty than CHE spent with wealthy individuals.

### 3.2 Data Collection

A complete collection of data from households is required to assess the frequency and severity of CHE. As a result, this study employed ZHHEUS data of 2014 [14]. The ZHHEUS was conducted in response to the need for accurate information on behavior when seeking healthcare, healthcare utilization, and healthcare spending at the level of households to guide future healthcare financing policies. The collection of data was concluded in February 2014, with a 99.5% response rate. For collecting data, the recall approach was employed with a 4‐week recall period. Questionnaires that were structured with a mix of open‐ and closed‐ended questions were used for the survey. In‐person interviews with respondents were used for administering the questionnaires. The survey's target population included all Zambian households in 10 provinces in 2014, except institutionalized population segments and diplomatic officials authorized to Zambia.

One of the primary advantages of family data from surveys overall, and the ZHHEUS in specific, is its national and sub‐populational representation. The survey employed a two‐stage stratified cluster sampling design. A two‐stage, categorized group sampling technique was used for the survey. The framework of sampling for the investigation was an inventory of the accepted list of regions (ARs) for the entire country. The region of study was divided into provinces, which were subdivided further into urban and rural areas. Provinces were stratified based on geographical and administrative subdivisions, whereas both urban and rural regions were stratified depending on socioeconomic fluctuation. The probability corresponding to the estimated size process was used in the initial phase to select ARs in every stratum. The systematic random sampling approach was employed in the second stage to select twenty families from every AR. During the data analysis process, this complicated design of sampling must be taken into consideration.

### 3.3 Data Analysis

The ZHHEUS gathered information that allowed individuals to be classified by social security employing a combination of direct (earnings, consumption, and expenses) and indirect (wealth) approaches. The population is split into quintiles, which separate people into five categories, with each category corresponding to 20% of the total number of people eligible for analysis. Every category has been categorized to indicate a socioeconomic section ranging from the lowest income bracket to the richest, utilizing both direct and indirect techniques. Quintiles are often characterized by earnings, consumption, expenses, and economic status based on survey data. The research examines the average OOP as a share of overall spending and individual spending across socioeconomic classes by using spending as an indicator of social security and defining quintiles as necessary because income is unpredictable for a large percentage of families in countries that are developing; it is an unreliable measure of living standards. Consumer spending and expenditure smoothing methods appear to be used by households as a cushion for income fluctuation. Consumer spending and expenditures, rather than income, are therefore a more trustworthy measure of the standard of living or welfare. There were two reasons for selecting spending excess as a social security indicator. To begin, although the consumer spending measure is more exhaustive for evaluating living standards, it involves an unbiased evaluation of the intrinsic worth of the products and services made by households. Second, overall spending by households acts as the basis for estimating CHE. Social Security is determined using household spending habits for uniformity across the study.

To show the negative nature of OOP, the mean OOP as a proportion of overall spending and as a proportion of personal spending is obtained based on every one of the expenditure quintiles. The ANOVA (analysis of variance) was conducted after calculating the means throughout the spending quintiles to figure out if any variations in their means were of statistical significance. According to the null hypothesis, the averages are statistically identical. If the difference in means between the socioeconomic categories is found to be of statistical significance, pairwise evaluations within the quintiles were carried out to find the unique economic status with significantly varying mean shares of OOP as a proportion of expenditure. Because OOP is a fixed price that does not fluctuate based on an individual's wealth, the assumption is that the higher the patients’ impoverishment, the bigger the proportion of overall and personal spending that OOP will consume.

Throughout the study, non‐food expenditure is used as a proxy for discretionary spending, according to the literature. Because food demand is presumed to be inelastic, examining OOP in terms of non‐food expenses as opposed to overall spending was suggested as a replacement measure of the capability to pay. The study offers predictions for both non‐food and overall expenditures for analysis purposes. After the adverse effect of OOP is established, the issue becomes whether such payments result in catastrophe incidents and, if so, what the extent of the incidents are. To track the full effect of the spending shock over time, such an analysis ought to be done, ideally with panel data. In the dearth of panel data, the research is dependent on a methodology introduced by Berki [16] and Wyszewianski [17], among others. This method assumes that expenditure beyond a particular threshold encompasses a material effect on a person's standard of living and is therefore devastating.

**FIGURE 1:**
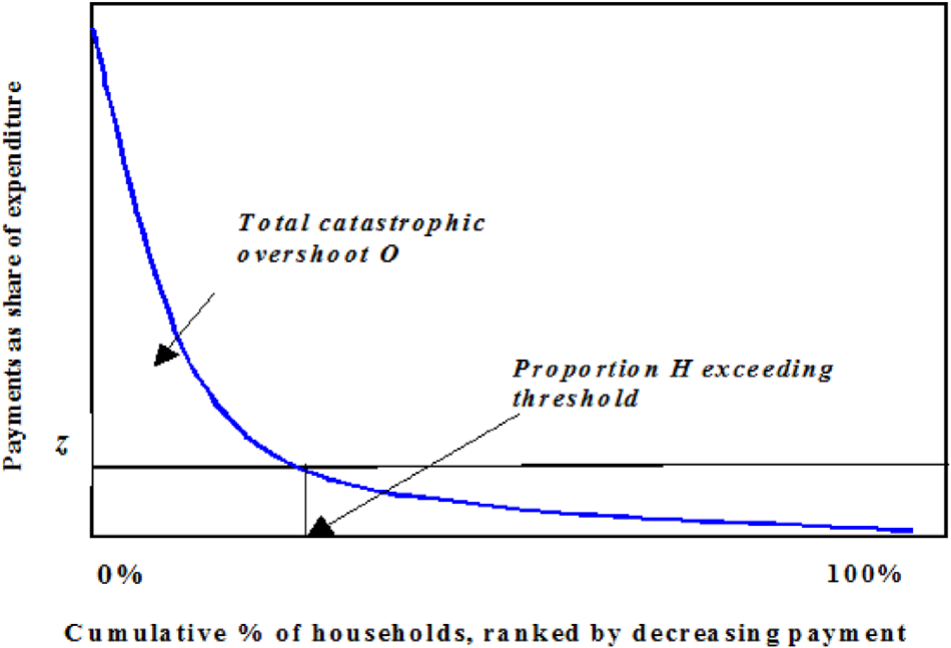
The budget share of health payments versus the cumulative percentage of individuals listed by reduced proportion of budget. *Source*: O'Donnell et al. [18].

OOP is defined as T to demonstrate the approach used to calculate the incidence of catastrophic payments. Total expenditure is denoted by the letter x, and non‐food expenditure is denoted by the letter f(x). Every household's OOP as a proportion of its overall and non‐food spending is represented by the ratios of T/x and T/f(x). According to the proportion of OOP overall and other than food spending, the families are organized into categories from lowest to biggest. Figure 1 depicts a cumulative percentage of families ranked by reducing the allocated fund's part, with the y‐axis representing the OOP proportion of spending. The probability of CHE for a particular limit, z, is a portion of the sample, H, with expenses for healthcare surpassing this level as a proportion of total spending. The disastrous overshoot, abbreviated as O, is an indicator of the number of disastrous transactions that surpass the z limit. The region situated above the limit and to the left of H represents O graphically. The disastrous overshoot is an indicator of the mean amount to which OOP as a percentage of total expenditure surpasses the limit, that is, the degree to which OOP occurrences surpass the disastrous level.

As another example of the computation of the incidence of CHE, the disastrous payments headcount (H) is illustrated arithmetically in Equation (1). The letter n represents the sample size. The measure $S_{i}$ returns a value of “1” if T/x and T/f(x) surpass each of their limits; alternatively, it returns a value of O. The frequency of CHE is represented by H, which is calculated by adding all incidents of S with the value 1; that is, where families’ portion of spending surpasses the limit, followed by determining the mean by splitting the result by the sample population size, n:

(1) $$H=\frac{1}{n}\;\sum_{i\;=\;1}^{n}S_{i}$$

Equation (2) illustrates a mathematical model of a person or household's overshoot, $S_{i}$, to display the CHE magnitude calculations. For families that have CHE, the value of their overshoot is the variance among the proportion of OOP in spending, T/x or T/f(x), and the limit, z, that is, OOP as a proportion of spending surpasses the limit; therefore, S requires the value of 1. For people not confronted with CHE, S is set to 0, and their overshoot has been set to 0:

(2) $$\;O_{i}=S_{i}\left(T/x_{i}\right)-z$$

Given that the value of the overshoot is O for every household in the data set, it is calculated as the average overshoot for the entire number of families within the group, as shown in the following equation:

(3) $$O=\frac{1}{n}\sum_{n}^{i\;=\;1}O_{i}$$

The O overshoot symbolizes the magnitude of CHE based on the population as a whole. The severity of CHE may be regarded independently for families that experienced CHE incidences. Equation (4) depicts the average beneficial overshoot (mean positive overshoot [MNPO]) when only CHE‐affected households are considered. It is determined by dividing the headcount (H) by the overshoot (O):

(4) MNPO=H/O

The MNPO allows you to see the overshoot, O, which appears in Equation (5) underneath as the employee numbers multiplied by the average beneficial overshoot. The connection suggests that the population's overall CHE magnitude (O) is determined by the product of the population's frequency of CHE (H) and the magnitude of the costs that individuals suffering from CHE encounter (MNPO):

(5) O=MNPO×H

The frequency and severity of CHE, symbolized by the initials H and O, at this stage, fail to account for the spread of CHE between families of differing socioeconomic status. Individuals with low incomes had a higher opportunity cost of healthcare expenses compared to the wealthy due to the presumption of diminishing marginal utility of revenue. Equity is put at risk in regions in which the poor have been disproportionately impacted by CHE. To evaluate the equity impacts, the investigation modifies the H and O computations to take into account the differential opportunity cost of healthcare expenditures among rich and poor people.

Calculating concentration indices for $S_{i}$ and $O_{i}$, symbolized $C_{S}$ and $C_{O}$, accordingly, is the initial step toward illustrating the differential opportunity cost of health spending. A concentration index, represented graphically as a concentration curve, measures the extent of socioeconomic disparity related to a variable. The concentration index $C_{S}$ discloses the level to which CHE occurrences are biased toward individuals with low income, whereas the concentration index $C_{O}$ provides the degree to which individuals with low income encounter a higher level of CHE compared to the wealthy. Unfavorable figures for $C_{S}$ as well as $C_{O}$ suggest the poor are more inclined to experience occurrences of disastrous payments, as well as the magnitude of these payments. The positive concentration index value suggests those with greater wealth have a greater frequency and severity of healthcare spending.

Once the two concentration indices $C_{S}$ and $C_{O}$ have been determined, the combination of them is then multiplied using the unweighted number of employees, H, and unadjusted overshoot, *O* to produce the adjusted number of employees, $H_{A}$, and adjusted overshoot, $O_{A}$. Equation (6) symbolizes the weighted number of employees, $H_{A}$, whereas Equation (7) symbolizes the adjusted overshoot, $O_{A}$:

(6) $$\;H_{A}=H\times \left(1-C_{s}\right)$$

(7) $$O_{A}=O\times \left(1-C_{O}\right)$$

If the poor face more frequent and severe disaster payments, $C_{s}$ and $C_{O}$ are negative, implying that $H_{A}$ is more valuable than H and $O_{A}$ is more valuable than O. Estimates and estimation errors were efficiently adjusted based on the selection design, and sample weights were used.

## 4 Results

### 4.1 Explanatory Data Analysis

OOP that families experience is caused by CHE. OOP is retrogressive in the manner that its policies disproportionately burden the less fortunate over those who are wealthy. Table 1 shows the mean payments for OOP as a percentage of overall and food‐unrelated spending throughout spending quintiles, from richest to poorest. The mean OOP of the most impoverished families is 0.5% of their overall expenditure, whereas the average of those with the greatest wealth is 0.06%. The poorest households account for 1.1% of OOP as a percentage of other than food spending, whereas those with the highest incomes account for 0.14%.

**TABLE 1:** Average out‐of‐pocket (OOP) as a percentage of overall and food‐unrelated spending throughout spending quintiles.

Average OOP as a percentage of overall spending			Average OOP as a percentage of food‐unrelated spending		
Spending quintile	Average (%)	Standard errors	Spending quintile	Average (%)	Standard errors
Richest	0.064	0.012	Richest	0.137	0.044
Second	0.058	0.007	Second	0.154	0.032
Middle	0.101	0.012	Middle	0.293	0.049
Fourth	0.158	0.031	Fourth	0.962	0.443
Poorest	0.501	0.098	Poorest	1.061	0.155

*Source*: 2014 ZHHEUS data.

Table 2 shows an ANOVA of the average shares of OOP in overall spending and the average shares of OOP in other than food spending to confirm that the variability in the averages throughout spending quintiles is of statistical significance. A one‐way ANOVA is used to compare the means of at least two unconnected groups that are independent. The variables that are dependent in this analysis are the proportion of OOP in the overall spending and other than food spending, whereas the autonomous categories, on the other hand, are the spending quintiles from richest to poorest. It is hypothesized that there is no statistically significant distinction among the averages among the groups that are independent. At the 1% level of significance, the null hypothesis is rejected for the average of OOP as a proportion of overall spending and the average of OOP as a proportion of non‐food spending. As a result, in these two cases, both of the autonomous groups are significantly dissimilar.

**TABLE 2:** Analysis of variance (ANOVA) in average out‐of‐pocket (OOP) as a proportion of overall and food‐unrelated spending throughout spending quintiles.

Average OOP as a proportion of overall spending				Average OOP as a percentage of food‐unrelated spending			
Source	Between groups	Within groups	Total	Source	Between groups	Within groups	Total
Sum of squares	0.145	127.025	127.170	Sum of squares	0.825	2132.065	2132.890
Degrees of freedom	4.000	59,181.000	59,185.000	Degrees of freedom	4.000	59,181.000	59,185.000
Mean square	0.036	0.002	0.002	Mean square	0.206	0.036	0.036
F	16.830			F	5.720		
Prob > F	0.0000			Prob > F	0.0001		

*Note*: Prob > $\chi^{2}\;$= 0.000; $\chi^{2}$ (4) = 1.0e + 05.

*Source*: 2014 ZHHEUS data.

### 4.2 Empirical Results

Upon establishing that a minimum of both of the spending categories were significantly dissimilar, a pairwise comparison was run to determine which categories differed most significantly. Tables 3 and 4 show pairwise contrasts of the average OOP as a proportion of overall spending and as an overall percentage of food‐unrelated spending. In regards to the average OOP as a proportion of overall spending, at the 5% level of significance, the lowest income earners category is significantly dissimilar (greater) compared to any of the remaining categories. At the 5% level of significance, pairwise analyses of the average of OOP healthcare transactions as a proportion of food‐unrelated spending reveal that the richest and poorest categories, the poorest and mid‐categories, the richest and fourth category, and the poorest and second category have statistically different means.

**TABLE 3:** Summary of pairwise contrasts.

OOP healthcare expenses as a proportion of overall spending			OOP healthcare expenses as a proportion of non‐food spending		
	Tukey			Tukey	
	Contrast	p‐value		Contrast	p‐value
Fourth vs. poorest	−0.003 (0.001)	0.000	Fourth vs. poorest	−0.002 (0.003)	0.882
Middle vs. poorest	−0.004 (0.001)	0.000	Middle vs. poorest	−0.007 (0.003)	0.030
Second vs. poorest	−0.004 (0.001)	0.000	Second vs. poorest	−0.009 (0.002)	0.003
Richest vs. poorest	−0.004 (0.001)	0.000	Richest vs. poorest	−0.009 (0.002)	0.001
Middle vs. fourth	0.000 (0.001)	0.939	Middle vs. fourth	−0.005 (0.002)	0.266
Second vs. fourth	−0.001 (0.001)	0.554	Second vs. fourth	−0.007 (0.002)	0.052
Richest vs. fourth	−0.001 (0.001)	0.555	Richest vs. fourth	−0.007 (0.002)	0.032
Second vs. middle	0.000 (0.001)	0.947	Second vs. middle	−0.002 (0.002)	0.956
Richest vs. middle	0.000 (0.001)	0.948	Richest vs. middle	−0.002 (0.002)	0.908
Richest vs. second	2.40E − 06 (0.001)	1.000	Richest vs. second	0.000 (0.002)	1.000

*Note*: Expenditure quintiles = 10; number of comparisons = 10; standard errors are in parenthesis.

Abbreviation: OOP, out‐of‐pocket.

*Source*: 2014 ZHHEUS data.

**TABLE 4:** Frequency and severity of disastrous expenses regarding overall and food‐unrelated spending in Zambia.

OOP healthcare expenses as a proportion of overall spending				OOP healthcare expenses as a proportion of food‐unrelated spending			
Catastrophic payments measure	Threshold budget share, *z*			Catastrophic payments measure	Threshold budget share, *z*		
	5%	10%	15%		20%	30%	40%
Overshoot (OS)	1.110	1.090	1.080	OS	1.310	1.280	1.260
Standard errors (Std errors)	1.020	1.020	1.020	Std errors	1.100	1.100	1.100
Head count (HC)	1.520	1.260	1.180	HC	1.330	1.240	1.190
Standard errors (Std errors)	1.040	1.020	1.020	Std errors	1.030	1.020	1.020
Mean positive overshoot (MNPO)	20.780	34.380	44.180	MNPO	93.390	117.200	134.820

Abbreviation: OOP, out‐of‐pocket.

*Source*: 2014 ZHHEUS data.

The preceding examination of OOP illustrates its retrogressive nature and raises the issue of the extent to which it could contribute to disastrous results for families. CHE is described as expenditure on healthcare that surpasses a certain percentage of overall or other than food spending. The reasoning behind assessing the intensity and frequency of CHE is that these kinds of charges compel families to decrease the purchase of additional products and services, ultimately reducing their living standards. Under the theory of decreasing marginal utility of revenue, poorer households’ catastrophic expenditures ought to be adjusted substantially higher compared to more wealthy families. Indeed, for families with limited resources, reckless spending has a greater likelihood of driving the family beneath the line of poverty. Thus, establishing fairness for healthcare funding and insurance coverage requires an investigation of the frequency and magnitude of disastrous settlements, as well as their socioeconomic distribution.

Table 4 highlights the frequency and severity of disastrous spending in terms of both overall and other‐than‐food spending, alongside healthcare settlements as a percentage of overall and other‐than‐food spending within different specified thresholds in Zambia. OOP as a percentage of total expenditure is measured at 5%, 10%, and 15% phases, whereas OOP as a percentage of non‐food spending is measured at 20%, 30%, and 40% levels. The proposed H value symbolizes the share of the overall families that experienced CHE at each of the established limits. The overshoot, denoted by the letter O, reflects the typical percentage of families that surpass the specified limit. The MNPO stands for the mean percentage of families that surpassed the provided limit, given that the criteria were to focus solely on the families that surpassed the specified limit. The overshoot, abbreviated as O, is the mean percentage of families that surpass the specified limit as a whole. The MNPO is established through the frequency of healthcare settlements that exceed the specified limit (headcount, H) alongside the severity associated with these purchases.

Utilizing 10% of OOP healthcare settlements as the proportion of the overall spending as the CHE limit, 1.26% of all families encounter CHE per year, resulting in households facing CHE surpassing the limit of 10% by a mean of 34%. On average, the overall number of families (which incorporates households that don't have CHE) surpasses the threshold of 10% by 1.09%. Although OOP is regarded as a proportion of other than food spending and the 40% threshold is applied, the prevalence of CHE was 1.19%; although families who experience CHE exceeded the 40% threshold by a mean of 135%, families as a whole surpass the limit by 1.26%.

**FIGURE 2:**
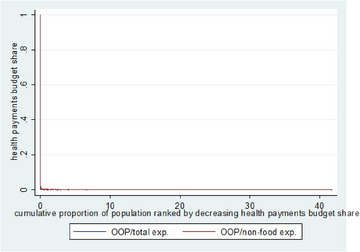
Healthcare expense budget proportion versus total household percentage ranked by reduced spending share. *Source*: 2014 ZHHEUS data.

Figure 2 illustrates the frequency and severity of CHE produced by Stata. The graph shows that the overshoot and number of employees are trivial, verifying the result that a large number of families are not subject to CHE.

The unweighted frequency of disastrous settlements, headcount, H, and the unweighted assessment of magnitude, overshoot, O, are based on the assumption that disastrous settlements possess a similar effect throughout social security levels. This presumption, nonetheless, has flaws because the opportunity cost of earnings is greater for households with lower incomes. As a consequence, if the theory of decreasing marginal utility based on earnings holds, CHE spent by lower income families ought to be adjusted substantially higher than CHE spent by households with greater wealth. Table 4 highlights both the adjusted headcount, $H_{A}$, and weighted overshoot, $O_{A}$, which influence the spread of CHE to the previously determined headcount, H, and overshoot, O. Table 5 summarizes the concentration indices $C_{S}$ and $C_{O}$ of the parameters S and O. The concentration indices $C_{S}$ and $C_{O}$ represent the socioeconomic breakdown of disastrous compensation incidences and intensity, respectively. The adjusted headcount ($H_{A}$) and adjusted overshoot ($O_{A}$) are determined using Equations (6) and (7).

**TABLE 5:** Frequency and severity of disastrous expenses in consideration of the socioeconomic pattern of catastrophic healthcare expenditures (CHE) in Zambia.

OOP healthcare expenses as a proportion of overall spending				OOP healthcare expenses as a proportion of food‐unrelated spending			
Catastrophic payment measure	Threshold budget share, *z*			Catastrophic payments measure	Threshold budget share, *z*		
	5%	10%	15%		20%	30%	40%
Concentration index, $C_{S}$	−0.288	−0.408	−0.493	Concentration index, $C_{S}$	−0.439	−0.487	−0.518
Rank‐weighted head count, $H_{A}$	0.66%	0.37%	0.27%	Rank‐weighted head count, $H_{A}$	0.48%	0.36%	0.29%
Concentration index, $C_{O}$	−0.616	−0.671	−0.702	Concentration index, $C_{O}$	−0.492	−0.495	−0.494
Rank‐weighted overshoot, $O_{A}$	0.17%	0.15%	0.13%	Rank‐weighted overshoot, $O_{A}$	0.46%	0.42%	0.39%

Abbreviation: OOP, out‐of‐pocket.

*Source*: 2014 ZHHEUS data.

If OOP healthcare expenses are taken as a proportion of the overall spending and employ a limit of 10%, the concentration indices $C_{S}$ was −0.41, whereas $C_{O}$ was −0.67. This implies that the fundamental factors are concentrated among those who are impoverished, given that the indices have negative values. This implies that the frequency and severity of CHE biases substantially toward households with lower incomes. At a 10% limit, the adjusted headcount ($H_{A}$) is 0.37, and the adjusted overshoot ($O_{A}$) is 0.15%. Utilizing a 40% threshold as well as payment for OOP as a proportion of other than food spending, the concentration indices $C_{S}$ is −0.52 and $C_{O}$ is −0.49%, leading to an adjusted headcount of 0.29% and adjusted overshoot of 0.39%. When the disproportionate impact of these factors on those who are impoverished is taken into account, it is unambiguous that a worse‐off image of CHE frequency and severity emerges.

## 5 Discussion

The CHE investigation determines its frequency and severity, in addition to the reality that those who are impoverished are more severely impacted by CHE. If the absolute value of the frequency of CHE across every one of the limits assessed is looked at, it is recognized that those figures are relatively small when compared with identical research in different nations. These findings support those of Akinkugbe et al. [19, 20] and Song et al. [11]. For example, Chen et al. [2] studied the level and distribution of CHE from 2013 to 2018 and discovered that (1) poor households were more likely to incur CHE than rich households, and (2) rural poor households were more likely to face CHE than urban poor households during the study period. Furthermore, a study of the disastrous and ruining outcomes of healthcare settlements in Uganda using household data from 2009/2010 found that 22.8% of families faced CHE at 10% OOP as a percentage of their overall spending [20]. At the 40% limit, the frequency of CHE was found to be 7% in Botswana and 1% in Lesotho [19].

Table 3 illustrates the retrogressive nature of OOP and raises the question of how far it can lead to disastrous consequences for families. Findings of Table 3 are consistent with those reported by Refs. [20, 21, 22, 23, 24, 25, 26]. This finding is consistent with the findings of Oyando et al. [25], which show that individuals aged 34–65, people living in cities, households headed by men, married households, employed households, and individuals working as self‐employed in the nonagricultural sector have a higher incidence and intensity of CHE. People over the age of 60, those living in rural areas, female‐headed households, unemployed households, and employers employing more than 10 people have the lowest incidence and intensity of CHE.

Table 5 summarizes the concentration indices, with results indicating that $C_{S}$ was −0.41 and $C_{O}$ was −0.67. This implies that the fundamental factors are concentrated among the poor, as the indices have negative values. This finding is consistent with Chen et al. [2] and Sataru et al. [13]. For example, Chen et al. [2] investigated the frequency and severity of CHE and used the concentration index to estimate the magnitude of disparities in CHE. Their study discovered that the frequency and severity of CHE increased between 2013 and 2018, with low‐income households having a higher concentration of CHE. Similarly, Sataru et al. [13] estimated the prevalence of catastrophic payments in Ghanaian households. The findings suggested that financial disasters were more concentrated among the poorest households, with significant disparities in incidence between the poorest and richest households.

## 6 Policy Recommendation

The policy implications of this study's findings highlight the importance of combining financial protection with an equal measure of success in providing equitable access to quality services. To advance UHC, the health system's revenue collection, pooling, and purchasing functions must be reformed. The sustainability of funding to the health sector resulting from revenue collection is a key issue in the UHC discussion, particularly in Zambia. External donors contribute significantly to the financing of the health system through development assistance. Although donor funding has helped to improve Zambian health outcomes, it is less reliable than domestic revenue sources and introduces vertical systems that are not always in sync with GRZ systems, resulting in significant inefficiencies. Private health contributions are the health system's least significant source of funding. Private contributions include voluntary prepayments and OOPs by individuals and businesses. Zambia's policy of providing free primary healthcare contributes to the relatively small share of PHE allocated to THE. Although this is a commendable policy for addressing equity in the use of health services, and it undoubtedly contributes to the low incidence and intensity of CHE, it is critical that issues concerning the policy's sustainability be addressed in order for it to be effective.

The low levels of PHE suggest that there is potential for increasing healthcare funding through private contributions. Private contributions to the health sector should not be collected through OOPs, as this would undermine the principle of equity in health financing and financial protection, which is currently protected by the free primary healthcare policy. Instead, the GRZ could increase private contributions to health financing by implementing mandatory prepayment in a social health insurance scheme. Social health insurance would increase the pool of funds available for purchasing health services, thereby improving financial security. In addition to the opportunity to leverage private resources through a mandatory prepayment scheme, the GRZ must continue to prioritize health in its budget allocation. Increasing GRZ allocation to health would thus advance the UHC goals. GGHE remains a critical source of funding for the health sector because it naturally functions as a risk pool and ensures effective cross‐subsidization. Zambia's tax code is progressive, which means that it is designed to promote equity. Increasing GRZ allocation to health would thus advance the UHC goals. Such an increase is largely dependent on the availability of fiscal space and the degree to which health is regarded as a priority in the national development agenda.

## 7 Conclusion

This study looked at the relationship between catastrophic healthcare costs and consumer financial protection in Zambia. The study's key finding is that the incidence and intensity of CHE are remarkably low when compared to similar studies conducted in other countries. Despite its low incidence and intensity, CHE distribution is skewed toward the poor. This means that the poor are more likely to experience CHE than the rich, and that the poor exceed catastrophic boundaries by a greater margin. This finding is consistent with the observation that OOPs consume more of the poor's income than the wealthy's. Similarly, the negative concentration index of the intensity of CHE at all thresholds indicates that the incidence and intensity of CHE are skewed toward the poor, implying that the poor bear a disproportionate burden of CHE compared to the wealthy. This finding supports the claim that the average OOPs as a share of household expenditure is significantly higher for the poor than for the rich. To the extent that CHE is a reliable indicator of financial protection, this is a promising finding for Zambia's path to UHC.

## Author Contributions

**MccPowell Fombang**: conceptualization, writing—original draft, formal analysis, writing—review and editing. **Richard Wamalwa Wanzala**: formal analysis, methodology, validation, writing—review and editing.

## Ethics Statement

We received ethical approval from the National Health Research Ethics Committee (of Stellenbosch University) with registration number REC‐050411‐032.

## Conflicts of Interest

The authors declare no conflicts of interest.

## Data Availability

The data that support the findings of this study are available in the Zambia Household Health Expenditure and Utilization Survey at https://www.dhsprogram.com/pubs/pdf/fr304/fr304.pdf. These data were derived from the following resources available in the public domain: Zambia Household Health Expenditure and Utilization Survey, https://www.dhsprogram.com/pubs/pdf/fr304/fr304.pdf.
